# Comment on “Predicting the mortality due to covid-19 by the next month for Italy, Iran and South Korea; a simulation study” 

**Published:** 2020

**Authors:** Golshan Mirmomeni, Arash Bayat

**Affiliations:** 1 *Student’s Research Committee, Ahvaz Jundishapur University of Medical Sciences, Ahvaz, Iran*; 2 *Hearing Research Center, Ahvaz Jundishapur University of Medical Sciences, Ahvaz, Iran *

**To The Editor**

We have read with great interest the study by Shojaee et al. (1) entitled “predicting the mortality due to COVID-19 by the next month for Italy, Iran and South Korea; a simulation study.” During this study, the authors precisely estimated the number of laboratory-confirmed COVID-19 patients and the rate of death among three countries in the next month using a “Poisson” distribution.

Poisson distribution is the common discrete probability distribution for the modeling of counts data. This type of distribution assumes an equi-dispersion of data (2). The equi-dispersion results when the variance and the mean are equal.

However, in real life, the populations are usually non-uniform (heterogeneous) and for several count data, the mean is not necessarily equal to that variance. This phenomenon is called as a problem of over-dispersion (3). Endo et al. (4) findings also indicated that the distribution of COVID-19 infection is highly over-dispersed.

It has been suggested that the most appropriate approach to deal with the problem of over-dispersion is using the “negative binomial” (NB) distribution. The NB distribution can be utilized for over-dispersed count data when the conditional variance is higher than the conditional mean (2). If the distribution of the outcome variable is over-dispersed, then the confidence intervals (CIs) for the negative binomial distribution are probably to be narrower than those derived from a Poisson distribution.

In conclusion, we suggest simulating the mortality rate in COVID-19 studies using the “negative binomial” distribution. This distribution could provide more flexible functional forms than Poison distribution to accommodate over-dispersion. 

## Conflict of interests

The authors declare that they have no conflict of interest.
