# Supplementary material for: Comment on “Predicting the mortality due to covid-19 by the next month for Italy, Iran and South Korea; a simulation study”
Source: Gastroenterol Hepatol Bed Bench. 2020 Autumn;13(4):421–2. (PMC7682968)
Supplement: Related texts

# Reply: Authors' responses to letter

**Published:** 2020

**Authors:** Sajad Shojaee, Mohamad Amin Pourhoseingholi

**Affiliations:** 1 * Gastroenterology and Liver Diseases Research Center, Research Institute for Gastroenterology and Liver Diseases, Shahid Beheshti University of Medical Sciences, Tehran, Iran*; 2 * Basic and Molecular Epidemiology of Gastrointestinal Disorders Research Center, Research Institute for Gastroenterology and Liver Diseases, Shahid Beheshti University of Medical Sciences, Tehran, Iran *

**To The Editor**

We are appreciate to Golshan Mirmomeni and Arash Bayat for reading our paper (1) with interest. 

As they mentioned in their letter, in the real life count data are usually non-uniform (heterogeneous) and in most cases the mean is not necessarily equal to that variance, which over-dispersion. We agree that negative binomial could be an appropriate approach to deal with the problem of over-dispersion. However, in our simulation, we believe the accuracy of model, despite of the problem of over-dispersion, is in a good level. In our study, main goal was to predict the number of confirmed and death cases in just short period of one month. In addition, we consider the items as an input data in a queue system by day-to-day, and according to a study of Bhat (2), the input distribution in the queue system has a Poisson distribution. We used this default and predicted new cases and mortality in the coming days. Now, it is worth mentioning that we have passed the forecast date mentioned in the study, after checking and verifying the predictions with the reality that happened in the surveyed countries (and reported as the official statistics of COVID-19 infection and mortality), the accuracy of our results will be confirmed. By the way, using the negative binomial approach could be useful for feature studies to predict the future behavior of COVID-19. 

Sincerely.

## References

[1] Shojaee S, Pourhoseingholi MA, Ashtari S, Vahedian-Azimi A, Asadzadeh-Aghdaei H, Zali MR (2020). Predicting the mortality due to Covid-19 by the next month for Italy, Iran and South Korea; a simulation study. Gastroenterol Hepatol Bed Bench.

[2] Bhat UN (2015). An introduction to queueing theory: modeling and analysis in applications.
